# Health care workers’ experiences during the COVID-19 pandemic: a scoping review

**DOI:** 10.1186/s12960-022-00724-1

**Published:** 2022-03-24

**Authors:** Souaad Chemali, Almudena Mari-Sáez, Charbel El Bcheraoui, Heide Weishaar

**Affiliations:** 1 Centre for International Health Protection, Robert Koch Institute, Nordufer 20, 13353 Berlin, Germany; 2 Evidence-Based Public Health, Centre for International Health Protection, Robert Koch Institute, Nordufer 20, 13353 Berlin, Germany

**Keywords:** Health care workers, Experiences, Resilience, Coping, COVID-19, Health systems, Pandemic

## Abstract

**Background:**

COVID-19 has challenged health systems worldwide, especially the health workforce, a pillar crucial for health systems resilience. Therefore, strengthening health system resilience can be informed by analyzing health care workers’ (HCWs) experiences and needs during pandemics. This review synthesizes qualitative studies published during the first year of the COVID-19 pandemic to identify factors affecting HCWs’ experiences and their support needs during the pandemic. This review was conducted using the Joanna Briggs Institute methodology for scoping reviews. A systematic search on PubMed was applied using controlled vocabularies. Only original studies presenting primary qualitative data were included.

**Results:**

161 papers that were published from the beginning of COVID-19 pandemic up until 28th March 2021 were included in the review. Findings were presented using the socio-ecological model as an analytical framework. At the individual level, the impact of the pandemic manifested on HCWs’ well-being, daily routine, professional and personal identity. At the interpersonal level, HCWs’ personal and professional relationships were identified as crucial. At the institutional level, decision-making processes, organizational aspects and availability of support emerged as important factors affecting HCWs’ experiences. At community level, community morale, norms, and public knowledge were of importance. Finally, at policy level, governmental support and response measures shaped HCWs’ experiences. The review identified a lack of studies which investigate other HCWs than doctors and nurses, HCWs in non-hospital settings, and HCWs in low- and lower middle income countries.

**Discussion:**

This review shows that the COVID-19 pandemic has challenged HCWs, with multiple contextual factors impacting their experiences and needs. To better understand HCWs’ experiences, comparative investigations are needed which analyze differences across as well as within countries, including differences at institutional, community, interpersonal and individual levels. Similarly, interventions aimed at supporting HCWs prior to, during and after pandemics need to consider HCWs’ circumstances.

**Conclusions:**

Following a context-sensitive approach to empowering HCWs that accounts for the multitude of aspects which influence their experiences could contribute to building a sustainable health workforce and strengthening health systems for future pandemics.

**Supplementary Information:**

The online version contains supplementary material available at 10.1186/s12960-022-00724-1.

## Introduction

The COVID-19 pandemic has put health systems worldwide under pressure and tested their resilience. The World Health Organization (WHO) acknowledges health workforce as one of the six building blocks of health systems [1]. Health care workers (HCWs) are key to a health system’s ability to respond to external shocks such as outbreaks and as first responders are often the hardest hit by these shocks [2]. Therefore, interventions supporting HCWs are key to strengthening health systems resilience (ibid). To develop effective interventions to support this group, a detailed understanding of how pandemics affect HCWs is needed.

Several recent reviews [3–27] focus on HCWs’ experiences during COVID-19 and the impact of the pandemic on HCWs’ well-being, including their mental health [3, 7, 8, 11–14, 16–27]. Most of these reviews refer to psychological scales measurements to provide quantifiable information on HCWs’ well-being and mental health [8, 13, 14, 19, 21–25, 28]. While useful in assessing the scale of the problem, such quantitative measures are insufficient in capturing the breadth of HCWs’ experiences and the factors that impact such experiences. The added value of qualitative studies is in understanding the complex experiences of HCWs during COVID-19 and the contextual factors that influence them [29].

This paper reviews qualitative studies published during the first year of the pandemic to investigate what is known about HCWs’ experiences during COVID-19 and the factors and support needs associated with those experiences. By presenting HCWs’ perspectives on the pandemic, the scoping review provides the much-needed evidence base for interventions that can help strengthen HCWs and alleviate the pressures they experience during pandemics.

## Methods

The review follows the Joanna Briggs Institute (JBI) process and guideline on conducting scoping reviews [30]. JBI updated guidelines identify scoping reviews as the most suitable choice to explore the breadth of literature on a topic, by mapping and summarizing available evidence [30]. Scoping reviews are also suitable to address knowledge gaps and provide insightful input for decision-making [30]. The review also applies the PRISMA checklist guidance on reporting literature reviews [31].

### Information sources

A systematic search was conducted on PubMed database between the 9th and 28th of March 2021.

### Search strategy

Drawing on Shaw et al. [32] and WHO [33], the search strategy used a controlled vocabulary of index terms including Medical Subject Headings (Mesh) of the keywords and synonyms “COVID-19”, “HCWs”, and “qualitative”. Keywords were combined using the Boolean operator “AND” (see Additional file 1).

### Eligibility criteria

The population of interest included all types of HCWs, independent of geography and settings. Only original studies were included in the review. Papers further had to (1) report primary qualitative data, (2) report on HCWs’ experiences and perceptions during COVID-19, and (3) be available as full texts in English, German, French, Spanish or Arabic, i.e., in a language that could be reviewed by one or several of the authors. Studies focusing solely on HCWs’ assessment of newly introduced modes of telemedicine during COVID-19 were excluded from the review as their clear emphasis on coping with technical challenges deviated from the review’s focus on HCWs’ personal and professional experiences during the pandemic.

### Selection process

The initial search yielded 3976 papers. All papers were screened and assessed against the eligibility criteria by one researcher (SC) to identify relevant studies. A random 25% sample of all papers was additionally screened by a second researcher (HW). Any uncertainty or inconsistency regarding inclusion were resolved by discussing the respective articles (*n* = 76) among the authors.

### Data collection process

Based on the research question, an initial data extraction form was developed, independently piloted on ten papers by SC and HW and finalised to include information on: (1) author(s), (2) year of publication, (3) type of HCW, (5) study design, (6) sample size, (7) topic of investigation, (8) data collection tool(s), (9) analytical approach, (10) period of data collection, (11) country, (12) income level according to World Bank [34], (13) context, and (14) main findings related to experiences, factors and support needs. Using the final extraction form, all articles were extracted by SC, with the exception of four German articles (which were extracted by HW), one Spanish and one French article (which were extracted by AMS). As far as applicable, the quality of the included articles was appraised using the JBI critical appraisal tool for qualitative research [35].

### Synthesis methods

The socio-ecological model originally developed by Brofenbrenner was adapted as a framework to analyze and present the findings [36–38]. The model aims to understand the interconnectedness and dynamics between personal and contextual factors in shaping human development and experiences [36, 38]. The model was chosen, because it accounts for the multifaceted interactions between individuals and their environment and is thus suited to capture the different dimensions of HCWs’ experiences, the factors associated with those experiences as well as the sources of support identified. The five socio-ecological levels (individual, interpersonal, institutional, community and policy) of the model served as a framework for analysis and were used to categorise the main themes that were identified in the scoping review as relevant to HCWs’ experiences. The process of identifying the sub-themes was conducted by SC using an excel extraction sheet, in which the main findings were captured and mapped against the socio-ecological framework.

## Results

### Study selection

**Fig. 1:**
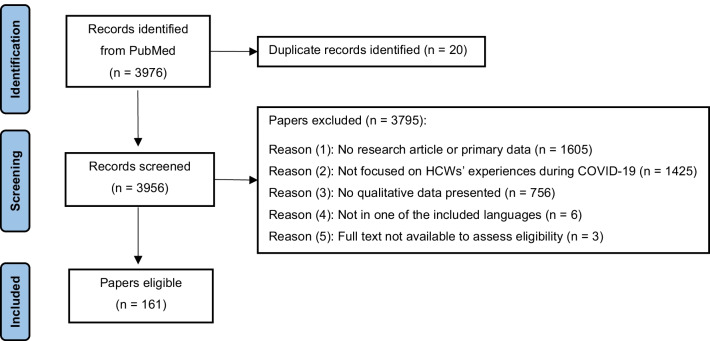
PRISMA flow diagram

The selection process and the number of papers found, screened and included are illustrated in a PRISMA flow diagram (Fig. 1). A total of 161 papers were included in the review (see Additional file 2). Table 1 lists the included studies based on study characteristics, including type of HCW, healthcare setting, income level of countries studied and data collection tools.

**Table 1:** Characteristics of included studies

Type of HCW	Frequency *n* (%)
Nurses	63 (39.13)
Doctors/physicians	24 (14.90)
Residents/students	7 (4.34)
Affiliated staff^a^	2 (1.24)
Pharmacists	1 (0.62)
Community health workers	1 (0.62)
Physical therapists	1 (0.62)
Midwives	1 (0.62)
Support staff working with people with intellectual disabilities	1 (0.62)
Home health care workers	1 (0.62)
Health and social care workers	1 (0.62)
Managerial staff	1 (0.62)
Multiple groups	57 (35.40)
**Frontline staff**	
Yes	71 (44.09)
No	39 (24.22)
Both	19 (11.80)
Not clear	32 (19.87)
**Healthcare setting**	
Hospitals	95 (59.00)
Primary care settings	9 (5.59)
Nursing homes	6 (3.72)
Home care	1 (0.62)
Residential disability support service	1 (0.62)
Community pharmacy	1 (0.62)
Prison	1 (0.62)
School	1 (0.62)
Independent abortion clinics	1 (0.62)
COVID-19 outpatient clinic	1 (0.62)
Multiple	28 (17.39)
Not clear	16 (9.93)
**World Bank income level of countries studied**	
High	98 (60.86)
Upper middle	44 (27.32)
Lower middle	10 (6.21)
Low	1 (0.62)
Not applicable	1 (0.62)
Multiple	7 (4.34)
**Data collection tools**	
Interviews	100 (62.11)
Open-ended questionnaires	40 (24.84)
Focus Groups	8 (4.96)
Social media, online platforms or recording systems submissions	5 (3.10)
Observations	1 (0.62)
Open reflections	1 (0.62)
Multiple	6 (3.72)

^a^Affiliated staff include paramedics, assistants, and technicians

### Study characteristics

Included papers investigated various types of HCWs. The most investigated type were nurses, followed by doctors/physicians. Medical and nursing students were also studied frequently, while only a small number of studies focused on other professions, e.g., community health workers, therapists and managerial staff. A third of all studies studied multiple HCWs, rather than targeting single professions. The majority of papers investigated so-called “frontline staff”, i.e., HCWs who engaged directly with patients who were suspected or confirmed to be infected with COVID-19. Fewer studies focused on non-frontline staff, and some explored both frontline and non-frontline staff.

Around two-thirds of all papers studied HCWs’ experiences in high-income countries, notably the USA, followed by the UK. Many papers also focused on HCWs in upper-middle income countries, with almost half of them conducted in China. Few papers investigated HCWs in lower-middle income countries, including India, Zimbabwe, Pakistan, Nigeria, and Senegal. Finally, one paper focused on HCWs in Ethiopia, a low-income country. A couple of studies presented data from multiple countries of different income levels, and one study investigating HCWs in Palestine could not be categorised. Overall, the USA was the most studied and China the second most studied geographical location (see Additional file 3). Hospitals were by far the most investigated healthcare settings, whereas outpatient settings, including primary care, pharmacies, homes care, nursing homes, healthcare facilities in prisons and schools as well as clinics, were investigated to a considerably lesser extent. Several studies covered more than one setting.

All studies applied a cross-sectional study design, with 54% published in 2020, and the remainder in 2021. A range of qualitative data collection methods were applied, with interviews being by far the most prominent one, followed by open-ended questionnaires. Focus groups and a few other methods including social media, online platforms or recording systems submissions, observations and open reflections were used with rare frequencies. The sample size in studies using interviews ranged between 6 and 450 interviewees. The sample size in studies using Focus Group Discussions (FGDs) ranged between 7 and 40 participants. Further information on the composition and context of the FGDs can be found in additional file 4. Several studies used multiple data collection tools. The majority of studies applied common analysis methods, including thematic and content analysis, with few using other specific approaches.

### Results of syntheses

An overview of the findings based on the socio-ecological framework is summarised in Table 2, which lists the main sub-themes identified under each socio-ecological level.

**Table 2:** Summary of findings

Socio-ecological framework of health care workers’ experiences during COVID-19		
Themes	Description	Sub-themes
Individual level	HCWs’ well-being, professional and personal identity, daily work–life routine	(-) The pandemic compromised HCWs’ physical and mental well-being (-) HCWs coped with the pandemic by employing diverse practices and activities (-) COVID-19 made some HCWs question their career choice (-) HCWs reported positive impact on their personal and professional identity particularly in the later pandemic stage (-) HCWs’ perceived well-being differed across settings, occupations and roles in the pandemic (-) HCWs’ experienced work–life imbalance (-) COVID-19 disrupted HCWs’ work routines (-) HCWs experienced negative financial effects
Interpersonal level	HCWs’ relationships with their private and professional environment	(-) HCWs’ were concerned with regard to virus transmission to their private context (-) HCWs introduced changes to their living situation in response to the pandemic (-) Interpersonal relationships were generally perceived as supportive by HCWs (-) Some HCWs felt shunned by family and friends (-) HCWs valued teamwork, but also experienced challenging collegial relationships (-) The pandemic compromised HCW-patient communication
Institutional level	Decision-making processes at work, organizational factors and availability of institutional support	(-) HCWs were discontent about institutional leadership (-) There was a perceived lack of institutional communication and organizational preparedness (-) HCWs experienced unfair allocation of work and roles in the workplace (-) There was dissatisfaction with institutional support and resources availability (-) HCWs advocated for training on health emergencies topics (-) Power hierarchies emerged as a factor influencing HCWs’ perceived sense of support
Community level	Morale, norms, and public knowledge and support	(-) HCWs’ perception of public support varied across roles and work settings (-) Ambivalence toward the “hero status” attributed to HCWs (-) HCWs experienced stigma with consequences on their personal lives (-) Public awareness of the dangers of COVID-19 was identified as an important factor impacting HCWs’ experiences (-) Online resources and facilitated information exchanges were perceived as useful by HCWs
Policy level	Perceived governmental response, support and the impact of its measures on HCWs	(-) HCWs’ voiced major dissatisfaction with governmental responses (-) Guidelines were perceived as contradictory (-) HCWs reported unequal distribution of governmental support across health facilities (-) Some governmental measures had a negative impact on HCWs’ personal and work lives (-) HCWs advocated for clear crisis communication, employees’ rights, and tailored pandemic preparedness and crisis management

### Individual level

At the individual level, HCWs’ experiences related to their well-being, professional and personal identity as well as daily work–life routine. In terms of well-being, HCWs reported negative impacts on their physical health (e.g., tiredness, discomfort, skin damage, sleep disorders) [39–55] and compromised mental health. The reported negative impact on mental health included increased levels of self-reported stress, depression, anxiety, fear, grief, guilt, anger, isolation, uncertainty and helplessness [39, 41, 43–47, 49–54, 56–123]. The reported reasons for HCWs’ reduced well-being included work-related factors, such as having to adhere to new requirements in the workplace, the lack and/or burden of using Personal Protective Equipment (PPE) [41, 44, 52, 63, 64, 78, 93, 124, 125], increased workload, lack of specialised knowledge and experience, concerns over delivering low quality of care [42, 44, 49, 52, 53, 63, 69, 70, 73, 74, 76, 78, 79, 83–86, 89, 90, 93, 94, 101, 103, 109, 125–140] and being confronted with ethical dilemmas [43, 72, 76, 78, 136, 141–145]. HCWs’ compromised psychological well-being was also triggered by extensive exposure to concerning information via the media and by the pressure that was experienced due to society and the media assigning HCWs hero status [53, 72, 81, 92, 97, 107, 139, 146]. Factors that were reported by HCWs as helping them cope with pressure comprised diverse self-care practices and personal activities, including but not limited to psychological techniques and lifestyle adjustments [47, 56, 64, 71, 72, 78, 90, 139, 147, 148] as well as religious practices [81, 112, 149].

Self-reported well-being differed across occupations, roles in the pandemic response and work settings. One study reported that HCWs working in respiratory, infection and emergency departments expressed more worries compared to HCWs who worked in other hospital wards [64]. Similarly, frontline HCWs seemed more likely to experience feelings of helplessness and guilt as they witnessed the worsening situation of COVID-19 patients, whereas non-frontline HCWs seemed to experience feelings of guilt due to not supporting their frontline colleagues [98]. HCWs with managerial responsibility reported heightened concern for their staff’s health [75, 110, 150]. HCWs working in nursing homes and home care reported feelings of being abandoned and not sufficiently recognised [75, 123, 144], while one study investigating HCWs responding to the pandemic in a slums-setting reported fear of violence [56].

HCWs reported that the pandemic impacted both positively and negatively on their professional and personal identity. While negative emotions were more dominant at the beginning of the pandemic, positive effects were reported to gradually develop after the initial pandemic phase and included an increased sense of motivation, purpose, meaningfulness, pride, resilience, problem-solving attitude, as well as professional and personal growth [43, 44, 47, 49–51, 63, 67–69, 71, 73–76, 78, 79, 87, 90–93, 98, 102, 104, 112, 114, 117–119, 122, 124, 131, 132, 143, 150–161]. Frontline staff reported particularly strong positive effects related to feelings of making a difference [69, 92]. On the other hand, some HCWs reported doubts with regard to their career choices and job dissatisfaction [40, 46, 59, 130]. Junior staff, assistant doctors and students often reported feelings of exclusion and concerns about the negative effects of the pandemic on their training [40, 162, 163]. Challenges with regard to their professional identity and a sense of failing their colleagues on the frontline were particularly reported by HCWs who had acquired COVID-19 themselves and experienced long COVID-19 [121, 160, 164]. HCWs who reached out to well-being support services expressed concern at being stigmatised [97].

HCWs reported a work–life imbalance [57, 97] as they had to adapt to the disruption of their usual work routine [59, 62, 131]. This disruption manifested in taking on different roles and responsibilities [39, 49, 67, 73, 83, 89, 94, 97, 110, 137, 139, 144, 151], increased or decreased workload pressure [85, 128, 130, 133] and sometimes redeployment [57, 155, 165]. HCWs also reported negative financial effects [59, 86, 166].

### Interpersonal level

The findings presented in this section relate to HCWs’ perceptions of their relationships in the private and professional environment during the pandemic and to the impact these relationships had on them. With regard to the home environment, HCWs’ concerns over being infected with COVID-19 and transmitting the virus to family members were identified in almost all studies [41, 44, 48, 51, 54, 56, 61, 68, 75, 77, 80, 85, 90, 128, 139, 160, 167–171]. HCWs living with children or elderly family members were particularly concerned [47, 65, 95, 97, 163, 172]. In some cases, HCWs reported that they had introduced changes to their living situation to protect their loved ones, with some deciding to move out to ensure physical distance and minimise the risk of transmission [39, 43, 44, 89, 105, 161]. Some HCWs reported sharing limited details about their COVID-19-related duties to decrease the anxiety and fear of their significant others [81]. While in several studies, interpersonal relationships were reported to cause concerns and worries, some study also identified interpersonal relationships and the subsequent emotional connectedness as a helpful resource [47, 173, 174] that could, for example, alleviate anxiety [64] or provide encouragement for working on the frontline [49, 106]. However, interpersonal relationships did not always have a supportive function, with some HCWs reporting being shunned by family and friends [66, 111, 175].

With regard to the work environment, relationships with colleagues were mainly described as supportive and empowering, with various studies reporting the value of teamwork during the pandemic [47, 51, 52, 67, 71, 77, 83, 91, 97, 98, 108, 134, 148, 151, 161]. Challenges with regard to collegial relationships included social distancing (which hindered HCWs’ interaction in the work place) [176] and working with colleagues one had never worked with before (causing a lack of familiarity with the work environment and difficulties to adapt) [79]. HCWs who worked in prisons reported interpersonal conflicts due to perceived increased authoritarian behaviour by security personnel that was perceived to manifest in arrogance and non-compliance with hygiene practices [88].

In terms of HCWs’ relationships with patients, many studies reported challenges in communicating with patients [50, 55, 126, 132, 133, 172]. This was attributed to the use of PPE during medical examinations and care and the reduction of face-to-face visits or a complete switch to telehealth [128, 139]. The changes in the relationships with patients varied according to the nature of work. Frontline HCWs, for example, reported challenges in caring for isolated patients [41, 43, 52, 148], whereas HCWs working in specific settings and occupational roles that required specific interpersonal skills faced other challenges. This was, for example, the case for HCWs working with people with intellectual disabilities, who found it challenging to explain COVID-19 measures to this group and also had to mitigate physical contact that was considered a significant part of their work [71]. For palliative care staff, the use of PPE and measures of social distancing were challenging to apply with regard to patients and family members [177]. Building relationships and providing appropriate emotional support was reported to be particularly challenging for mental health and palliative care professionals supporting vulnerable adults or children [117]. Challenges for health and social care professionals were associated with virtual consultations and more difficult conversations [117]. Physicians reported particular frustration with remote monitoring of chronic diseases when caring for low-income, rural, and/or elderly patients [169]. Having to adjust, and compromise on, the relationships with patients caused concerns about the quality of care, which in turn, was reported to impact negatively on HCWs’ professional identity and emotional well-being.

### Institutional level

This section presents HCWs’ perceptions of decision-making processes in the work setting, organizational factors and availability of institutional support.

With regard to decision-making, a small number of studies reported HCWs’ trust in the institutions they worked in [143, 172], while the majority of studies revealed discontent about institutional leadership and feelings of exclusion from decision-making processes [65, 178]. More specifically, HCWs reported a lack of clear communication and coordination [41, 70, 144, 148, 179] and a wish to be provided with the rationales behind management decisions and to be included in recovery phase planning [48]. They perceived centralised decision-making processes as unfamiliar and restrictive [150]. Instead, HCWs endorsed de-centralised and participatory approaches to communication and decision-making [56]. Emergency and critical care physicians suggested to include bioethicists as part of the decision-making on triaging scarce critical resources [126]. Studies of both hospital and primary care settings reported perceived disconnectedness and poor collaboration between managerial, administrative and clinical staff, which was a contributing factor to burnout among HCWs [60, 83, 149, 169, 180–182]. Dissatisfaction with communication also related to constantly changing protocols, which were perceived as highly burdening and frustrating, creating ambiguity and negatively affecting HCWs’ work performance [44, 55, 59, 78, 112, 183].

In terms of organizational factors, many HCWs reported a perceived lack of organizational preparedness and poor organization of care [60, 65, 120, 179]. Changes in the organization of care were perceived as chaotic, especially at the beginning of the pandemic, and changes in roles and responsibilities and role allocation were perceived as unfair and unsatisfying [72, 97]. Only in one study, changes in work organisation were perceived positively, with nurses reporting satisfaction with an improved nurse–patient ratio resulting from organisational changes [52]. Overall, frontline HCWs advocated for more stability in team structure to ensure familiarity and consistency at work [47, 66, 72, 114, 116]. HCWs appreciated multidisciplinary teams, despite challenges with regard to achieving rapid and efficient collaboration between members from different departments [41, 143, 152].

Regarding institutional support, in some instances, psychological, managerial, material and technical support was positively acknowledged, while the majority of studies reported HCWs’ dissatisfaction with the support provided by the institution they worked in [46, 48, 73, 84, 92, 97, 114, 139, 144, 174, 184]. Across studies, a lack of equipment, including the unavailability of suitable PPEs, was one of the most prominent critiques, especially in the initial phase the pandemic [41, 46, 54, 55, 61, 69, 70, 72, 73, 81, 84, 85, 96, 97, 111, 118, 144, 147, 168]. In one study of a rural nursing home, HCWs reported being illegally required to treat COVID-19 patients without adequate PPE [39]. Specialised physicians, such as radiologists, for example, reported that PPE were prioritised for COVID-19 ward workers [65]. In another instance, HCWs reported that they had taken care of their own mask supply [113]. Insufficient equipment and the subsequent lack of protection induced fear and anxiety regarding one’s personal safety [64, 87]. HCWs also reported inadequate human resources, which had consequences on increased workload [44, 46, 54, 69, 75, 85]. Dissatisfaction with limited infrastructure was reported overall and across settings, but specific limitations were particularly relevant in certain contexts [116]. HCWs in low resource settings, including Pakistan, Zimbabwe and India, reported worsening conditions regarding infrastructure, characterised by a lack of water supply and ventilation, poor conditions of isolation wards and lack of quality rest areas for staff [41, 58, 84]. Despite adaptive interventions aimed at shifting service delivery to outdoors, procedures such as patient registration and laboratory work took place in poorly ventilated rooms [56]. Technical support such as the accessibility to specialised knowledge and availability of training were identified by HCWs as an important resource that required strengthening. They advocated for better “tailor-made” trainings in emergency preparedness and response, crisis management, PPE use and infection control [41, 52, 61, 68, 73, 127, 144]. HCWs argued that the availability of such training would improve their sense of control in health emergencies, while a lack of training compromised their confidence in their ability to provide quality healthcare [47, 134].

Structural factors such as power hierarchies and inequalities played a role in HCWs’ perceived sense of institutional support amidst the quick changes in their institutions. Such factors were particularly mentioned in studies investigating nurses who reported dissatisfaction over doctors’ dominance and discrimination in obtaining PPE [54] as well as unfairness in work allocation [72, 184]. They also perceived ambiguity in roles and responsibilities between nurses and doctors [101]. A low sense of institutional support was also reported by other HCWs. Junior medical staff and administrative staff reported feeling exposed to unacceptable risks of infection and a lack of recognition by their institution [139]. Staff in non‐clinical roles, non-frontline staff, staff working from home, acute physicians and those on short time contracts felt less supported and less recognised compared to colleagues on the frontline [48, 139].

### Community level

This level entails how morale and norms, as well as public knowledge relate to HCWs’ experiences in the pandemic. On the positive side, societal morale and norms were perceived as enhancing supportive attitudes among the public toward HCWs and triggering community initiatives that supported HCWs in both emotional and material ways [47, 78, 92, 108, 140, 147]. This supportive element was especially experienced by frontline HCWs, who felt valued, appreciated and empowered by their communities. HCWs’ reaction to the hero status that was assigned to them was ambivalent [146, 185]. In response to this status attribution, HCWs reported a sense of pressure to be on the frontline and to work beyond their regular work schedule [51]. With community support being perceived as clearly focusing on hospital frontline staff, HCWs working from home, in nursing homes, home care and non-frontline facilities and wards perceived less public support [139] and appreciation [85, 144]. One study highlighted that HCWs did not benefit from this form of public praise but preferred an appreciation in the form of tangible and financial resources instead [160].

A clear negative aspect of social norms manifested in the stigmatisation and negative judgment by community members [72, 100, 106, 186, 187], who avoided contact with HCWs based on the perceptions that they were virus carriers and spreaders [43, 68, 92, 111]. Such discrimination had negative consequences with regard to HCWs’ personal lives, including lack of access to public transportation, supermarkets, childcare and other public services [65, 80, 107]. Chinese HCWs working abroad reported bullying due to others perceiving and labeling COVID-19 as the ‘Chinese virus’ [77]. Negative judgment was mainly reported in studies on nurses**.** In a study of a COVID-19-designated hospital, frontline nurses reported unusually strict social standards directed solely at them [122]. In a comparative study of nursing homes in four countries, geriatric nurses reported social stigma toward their profession, which the society perceive not worth of respect [75].

Beyond social norms, studies identified the level of public awareness, knowledge and compliance as important determinants of HCWs’ experiences and emotional well-being [147]. For example, a lack of compliance with social distancing and other preventive measures was reported to induce feelings of betrayal, anger and anxiety among HCWs [41, 80, 81, 111, 188]. The dissemination of false information and rumors and their negative influence on knowledge and compliance was also reported with anger by HCWs in general [58], an in particular by those who worked closely with local communities [129]. Online resources and voluntary groups facilitated information exchange and knowledge transfer, factors which were valued by HCWs as an important source of information and support [131, 189].

### Policy level

Findings presented here include HCWs’ perceptions of governmental responses, governmental support and the impact of governmental measures on their professional and private situation. In a small number of studies, HCWs expressed confidence in their government’s ability to respond to the pandemic and satisfaction with governmental compensation [45, 47]. In most cases, however, HCWs expressed dissatisfactions with the governmental response, particularly with the lack of health system organisation, the lack of a coordinated, unified response and the failure to follow an evidence-based approach to policy making. HCWs also perceived governmental guidelines as chaotic, confusing and even contradicting [61, 85, 86, 115, 117, 118, 120, 123, 147, 160, 182, 190]. In one study, inadequate staffing was directly attributed to inadequate governmental funding decisions [191]. Many studies reported that HCWs had a sense of being failed by their governments [60, 100, 191], with non-frontline staff, notably HCWs working with the disabled [71, 181], the elderly [39, 75, 123, 151] or in home-based care [58], being particularly likely to voice feelings of being forgotten, deprioritised, invisible, less recognised and less valued by their governments. Care home staff perceived governmental support to be unequally distributed across health facilities and as being focused solely on public institutions, which prevented them from receiving state benefits [149].

Measures and regulations imposed at the governmental level had a considerable impact on HCWs’ professional as well as personal experiences. In nursing homes, HCWs perceived governmental regulations such as visiting restrictions as particularly challenging and complained that rules had not been designed or implemented with consideration to individual cases [62]. The imposed rules burdened them with additional administrative tasks and forced them to compromise on the quality of care, resulting in moral distress [62]. In abortion clinics, HCWs expressed concerns about their services being classed as non-essential services during the early stages of the pandemic [190]. Governmental policies also had impacts on HCWs personally. For example, the closure of childcare negatively impacted HCWs’ ability to balance personal and private roles and commitments. National lockdowns which restricted travel made it harder for HCWs to get to work or to see their families, especially in places with low political stability [95]. The de-escalation of measures, notably the opening of airports, was perceived as betrayal by HCWs who felt they bore the burden of increased COVID-19 incidences resulting from de-escalation strategies [111].

HCWs identified clear and consistent governmental crisis communication [97, 126], better employees’ rights and salaries, and tailored pandemic preparedness and crisis management policies that considered different healthcare settings and HCWs’ needs [43, 64, 81, 101, 124, 160, 167, 169, 188, 192, 193] as important areas for improvement. HCWs in primary care advocated for strengthened primary health care, improved public health education [45, 130] and a multi-sectoral approach in pandemic management [129].

## Discussion

Our scoping review of HCWs’ experiences, support needs and factors that influence these experiences during COVID-19 shows that HCWs were affected at individual, interpersonal, institutional, community and policy levels. It also highlights that certain experiences can have disruptive effects on HCWs’ personal and professional lives, and thus identifies problems which need to be addressed and areas that could be strengthened to support HCWs during pandemics.

To the best of our knowledge, our review is the first to provide a comprehensive account of HCWs’ experiences during COVID-19 across contexts. By applying an exploratory angle and focusing on existing qualitative studies, the review does not only provide a rich description of the situation of HCWs but also develops an in-depth analysis of the contextual multilevel factors which impact on HCWs’ experiences.

Our scoping review shows that, while studies on HCWs’ experiences in low resource settings are scarce, the few studies that exist and the comparison with other studies point towards setting-specific experiences and challenges. We thus argue that understanding HCWs’ experiences requires comparative investigations, which not only take countries’ income levels into account but also other contextual differences. For example, in our analysis, we identify particular challenges experienced by HCWs working in urban slums and places with limited infrastructure and low political stability. Similarly, in a recent short communication in Social Science & Medicine, Smith [194] presents a case study on the particular challenges of midwives in resource-poor rural Indonesia at the start of the pandemic, highlighting increased risks and intra-country health system inequalities. Contextual intra-country differences in HCWs’ experiences also manifest at institutional level. For example, the review suggests that HCWs who work in non-hospital settings, such as primary care services, nursing homes, home based care or disability services, experienced particular challenges and felt less recognized in relation to hospital-based HCWs. In a similar vein, HCWs working in care homes felt that as state support was not equally distributed, those working in public institutions had better chances to benefit from state support.

The review highlights that occupational hierarchies play a crucial role in HCWs’ work-related experiences. Our analysis suggests that existing occupational hierarchies seem to increase or be exposed during pandemics and that occupation is a structural factor in shaping HCWs’ experiences. The review thus highlights the important role that institutions and employers play in pandemics and is in line with the growing body of evidence that associates HCWs’ well-being during COVID-19 with their occupational role [195] and the availability of institutional support [195, 196]. The findings suggest that to address institutional differences and ensure the provision of needs-based support to all groups of HCWs, non-hierarchical and participative processes of decision-making are crucial.

Another contextual factor affecting HCWs’ experiences are their communities. While the majority of HCWs experience emotional and material support from their community, some also feel pressure by the expectations they are confronted with. The most prominent example of such perceived pressure is the ambivalence that was reported with regard to the assignment of a hero status to HCWs. On the one hand, this attribution meant that HCWs felt recognized and appreciated by their communities. On the other hand, it led to HCWs feeling pressured to work without respecting their own limits and taking care of themselves.

This scoping review points towards a number of research gaps, which, if addressed, could help to hone interventions to support HCWs and improve health system performance and resilience.

First, the majority of existing qualitative studies investigate nurses’ and doctors’ experiences during COVID-19. Given that other types of HCWs play an equally important role in pandemic responses, future research on HCWs’ experiences in pandemics should aim for more diversity and help to tease out the specific challenges and needs of different types of HCWs. Investigating different types of HCWs could inform and facilitate the development of tailored solutions and provide need-based support.

Second, the majority of studies on HCWs’ experiences focus on hospital settings. This is not surprising considering that the bulk of societal and political attention during COVID-19 has been on the provision of acute, hospital-based care. The review thus highlights a gap with regard to research on HCWs in settings which might be considered less affected and neglected but which might, in fact, be severely collaterally affected during pandemics, such as primary health centers, care homes and home-based care. It also indicates that research which compares HCWs’ experiences across levels of care can help to tease out differences and identify specific challenges and needs.

Third, the review highlights the predominance of cross-sectional studies. In fact, we were unable to identify any longitudinal studies of HCWs’ experiences during COVID-19. A possible reason for the lack of longitudinal research is the relatively short time that has passed since the start of the pandemic which might have made it difficult to complete longitudinal qualitative studies. Yet, given the dynamics and extended duration of the pandemic, and knowledge about the impact of persistent stress on an individual’s health and well-being [197–200], longitudinal studies on HCWs’ experiences during COVID-19 would provide added value and allow an analysis across different stages of the pandemic as well as post-pandemic times. In our review, three differences in HCWs’ experiences across the phases of the pandemic were observed. The first one is on the individual level, reflecting the dominance of the negative emotions at the initial phase of the pandemic, which was gradually followed by increased reporting of the positive impact on HCWs’ personal and professional identity. The two other differences were on the institutional level, referring to the dissatisfaction over the lack of equipment and organization of care, mainly observed at the initial pandemic phase. Further comparative analysis of changes in HCWs’ experiences over the course of a pandemic is an interesting and important topic for future research, which could also map HCWs’ experiences against hospital capacities, availability of vaccines and tests as well as changes in pandemic restrictions. Such comparative analysis can inform the development of suitable policy level interventions accounting for HCWs’ experiences at different pandemic stages, from preparedness to initial response and recovery.

Finally, the majority of studies included in the review were conducted in the Northern hemisphere, revealing a gap in understanding the reality of HCWs in low- and lower middle income countries. Ensuring diversity in geographies and including resource-poor settings in research on HCWs would help gain a better contextual understanding, contribute to strengthening pandemic preparedness in settings, where the need is greatest, and facilitate knowledge transfer between the global North and South. While further research can help to increase our understanding of HCWs’ experiences during pandemics, this scoping review establishes a first basis for the evaluation and improvement of interventions aimed at supporting HCWs prior to, during and after COVID-19. A key finding of our analysis to strengthen HCWs’ resilience are the interdependencies of factors across the five levels of the socio-ecological model. For example, institutional, community or policy level factors (such as dissatisfaction with decision-making processes, public non-compliance or failures in pandemic management) can have a negative impact on HCWs at interpersonal and individual levels by impacting on their professional relationships, mental health or work performance. Similarly, policy, community or institutional level factors (such as adequate policy measures, appreciation within the community and the provision of PPE and other equipment) can act as protective factors for HCWs’ well-being. In line with the social support literature [201], interpersonal relationships were identified as a key factor in shaping HCWs’ experiences. The identification of the inter-dependencies between factors affecting HCWs during pandemics further highlights that health systems are severely impacted by factors outside the health systems’ control. Previous scholars have recognized the embeddedness of health systems within, and their constant interaction with, their socio-economic and political environment [202]. Previous literature, however, also shows that interventions tackling distress of HCWs have largely focused on individual level factors, e.g., on interventions aimed at relieving psychological symptoms, rather than on contextual factors [16]. To strengthen HCWs and empower them to deal with pandemics, the contextual factors that affect their situation during pandemics need to be acknowledged and interventions need to follow a multi-component approach, taking the multitude of aspects and circumstances into account which impact on HCWs’ experiences.

### Limitations and strengths

Our scoping review comes with a number of limitations. First, due to resource constraints, the search was conducted using only one database. The authors acknowledge that running the search strategy on other search engines could have resulted in additional interesting studies to be reviewed. To mitigate any weaknesses, extensive efforts were made to build a strong search string by reviewing previous peer-reviewed publications as well as available resources from recognized public health institutions. Considering the high numbers of studies identified, it can be, however, assumed that the search strategy and review led to valid conclusions. Second, the review excluded non-original publications. While other types of publications could have provided additional data and perspectives on HCWs’ experiences, we decided to limit our review to original, peer-reviewed research articles to ensure quality. Third, the review excluded studies on other pandemics, which could have provided further insights into HCWs’ experiences during health crises. Given the limited resources available to the research project, it was decided to focus only on COVID-19 to accommodate a larger target group of all types of HCWs and a variety of geographical locations and healthcare settings. Furthermore, it can be argued that previous pandemics did not reach the magnitude of COVID-19 and did not lead to similar responses. With the review looking at the burden of COVID-19 as a stressor, it can be assumed that the more important the stressor, the more interesting the results. Therefore, the burdens and the way in which HCWs dealt with these burdens would be particularly augmented with regard to COVID-19, making it a suitable focus example to investigate HCWs’ experiences in health crises. The authors acknowledge that during other pandemics HCWs’ experiences might differ and be less pronounced, yet this review has addressed stressors and ways of supporting HCWs that could also inform future health crises. In our view, a major strength of the review is that is does not apply any limitation in terms of the types of HCWs, the geographical locations or the healthcare settings included. This approach did not only allow us to review a wide range of literature on an expanding area of knowledge [30], but to appropriately investigate HCWs’ experiences during a public health emergency of international concern that affects countries across the globe. Providing detailed information about the contexts in which HCWs were studied, allowed us to shed light on the contextual factors affecting HCWs’ experiences.

### Implications for policy and practice

Areas of future interventions that improve HCWs’ resilience at individual level could aim towards alleviating stress and responding to their specific needs during pandemics, in line with encouraging self-care activities that can foster personal psychological resilience. Beyond that, accounting for the context when designing and implementing interventions is crucial. This can be done by addressing the circumstances HCWs live and work in, referred to in German-speaking countries as “Verhältnisprävention”, i.e., prevention through tackling living and working conditions. Respective interventions should tackle all levels outlined in the socio-ecological model, applying a systems approach. At the interpersonal level, creating a positive work environment in times of crises that is supportive of uninterrupted and efficient communication among HCWs and between HCWs and patients is important. In addition, interpersonal support, e.g., by family and friends could be facilitated. At institutional level, organizational change should consider transparent and participatory decision making and responsible planning of resources availability and allocation. At community level, tracing rumors and misinformation during health emergencies is crucial, as well as advocating for accountable journalism and community initiatives that support HCWs in times of crisis. At policy level, pandemic regulations need to account for their consequences on HCWs’ work situations and personal lives. Governmental policies and guidelines should build on scientific evidence and take into account the situations and lived experiences of HCWs across all levels of care.

## Conclusions

This scoping review of existing qualitative research on HCWs’ experiences during COVID-19 sheds light on the impact of a major pandemic on the health workforce, a key pillar of health systems. By identifying key drawbacks, strengths that can be built upon, and crucial entry-points for interventions, the review can inform strategies towards strengthening HCWs and improving their experiences. Following a systems approach which takes the five socio-ecological levels into account is crucial for the development of context-sensitive strategies to support HCWs prior to, during and after pandemics. This in turn can contribute to building a sustainable health workforce and to strengthening and better preparing health systems for future pandemics.

## Supplementary Information

**Additional file 1: Table S1.** Search strategy. The document includes the search strings for the review.

**Additional file 2: Table S2.** List of included papers. The file lists the 161 included papers, detailing the title, authors, publication year and DOI link.

**Additional file 3: Table S3.** List of countries studied. The file includes a table listing the countries in which the included studies were conducted according to frequency.

**Additional file 4: Table S4.** Detailed information on FGDs. This document provides information extracted from studies that used FGDs as a qualitative data collection tool. The table lists the overall number of focus group discussion’s participants in each of those studies, the number of FGDs per study, whether FGDs were conducted online or offline, the type of study participants, and any other information on the methods that could be extracted.

## Data Availability

All data generated during this study are included in this published article and its supplementary information files, except for a detailed extraction sheet for all studies included, which is available from the corresponding author upon request.

## Abbreviations

HCWs: Health care workers
JBI: Joanna Briggs Institute
FGDs: Focus Groups Discussions
PPE: Personal Protective Equipment
WHO: World Health Organization
