# Supplementary material for: Health care workers’ experiences during the COVID-19 pandemic: a scoping review
Source: Hum Resour Health. 2022 Mar 24;20:27. doi: 10.1186/s12960-022-00724-1 (PMC8943506; doi:10.1186/s12960-022-00724-1)
Supplement: Supplementary file 1 — Additional file 1: Table S1. Search strategy. The document includes the search strings for the review. [file 12960_2022_724_MOESM1_ESM.docx]

**Health Care Workers’ experiences during the COVID-19 Pandemic: a scoping review**

**Additional file (1): The search strategy**

The search string for “COVID-19” was taken from the Robert Koch Institute Library; the search string for “HCWs” was adapted from WHO (2016); the search strategy for “qualitative research” was adapted from a search string developed by Shaw et al (2004).

("Severe Acute Respiratory Syndrome Coronavirus 2" [Supplementary Concept] OR "COVID-19" [Supplementary Concept] OR "covid 19 diagnostic testing" [Supplementary Concept] OR "covid 19 drug treatment" [Supplementary Concept] OR "covid 19 serotherapy"[Supplementary Concept] OR "covid 19 vaccine" [Supplementary Concept] OR "Severe Acute Respiratory Syndrome Coronavirus 2"[tiab] OR ncov*[tiab] OR covid*[tiab] OR sars-cov-2[tiab] OR "sars cov 2"[tiab] OR "SARS Coronavirus 2"[tiab] OR "Severe Acute Respiratory Syndrome CoV 2"[tiab] OR "Wuhan coronavirus"[tiab] OR "Wuhan seafood market pneumonia virus"[tiab] OR "SARS2"[tiab] OR "2019-nCoV"[tiab] OR "hcov-19"[tiab] OR „novel 2019 coronavirus"[tiab] OR "2019 novel coronavirus*"[tiab] OR „novel coronavirus 2019*"[tiab] OR "2019 novel human coronavirus*"[tiab] OR „human coronavirus 2019"[tiab] OR "coronavirus disease-19"[tiab] OR "corona virus disease-19"[tiab] OR "coronavirus disease 2019"[tiab] OR "corona virus disease 2019"[tiab] OR "2019 coronavirus disease"[tiab] OR "2019 corona virus disease"[tiab] OR „novel coronavirus disease 2019"[tiab] OR „novel coronavirus infection 2019"[tiab] OR "new coronavirus*"[tiab] OR "coronavirus outbreak"[tiab] OR "coronavirus epidemic"[tiab] OR "coronavirus pandemic"[tiab] OR "pandemic of coronavirus"[tiab]) AND ("2019/12/01"[PDAT] : "2099/12/31"[PDAT])

AND

("Health Personnel" [MeSH] OR "health personnel" [all fields] OR "healthcare personnel"[all fields] OR "health care personnel"[all fields] OR "health worker"[all fields] OR "health workers"[all fields] OR "healthcare worker"[all fields] OR "healthcare workers"[all fields] OR "health care worker"[all fields] OR "healthcare provider"[all fields] OR "healthcare providers"[all fields] OR "health care provider"[all fields] OR "health practitioners"[all fields] OR "healthcare practitioner"[all fields] OR "health care practitioner"[all fields] OR "health care practitioners"[all fields] OR "health employee"[all fields] OR "health employees"[all fields] OR "medical staff"[all fields] OR doctor[all fields] OR doctors[all fields] OR physician*[all fields] OR ("allied health"[all fields] AND ("staff"[all fields] OR personnel[all fields])) OR paramedics*[all fields] OR "nursing staff"[all fields] OR nurse[all fields] OR nurses[all fields] OR "nursing auxiliary"[all fields] OR "hospital personnel"[all fields] OR "hospital staff"[all fields] OR "hospital worker"[all fields] OR "hospital workers"[all fields])

AND

("qualitative research"[MeSH Terms] OR "nursing methodology research"[MeSH Terms] OR "questionnaires"[All Fields] OR "attitude"[MeSH Terms] OR "focus groups"[MeSH Terms] OR "discourse analysis"[All Fields] OR "content analysis"[All Fields] OR "ethnographic research"[All Fields] OR "ethnological research"[All Fields] OR "ethnonursing research"[All Fields] OR "constant comparative methods"[All Fields] OR "qualitative validity"[All Fields] OR "purposive sample"[All Fields] OR "observational method*"[All Fields] OR "field stud*"[All Fields] OR "theoretical sampl*"[All Fields] OR "phenomenology"[All Fields] OR "phenomenological research"[All Fields] OR "life experience*"[All Fields] OR "cluster sampl*"[All Fields] OR "ethnonursing"[All Fields] OR "ethnograph*"[All Fields] OR "phenomenol*"[All Fields] OR "grounded theory"[MeSH Terms] OR "grounded theor*"[All Fields] OR "grounded study"[All Fields] OR "grounded studies"[All Fields] OR "grounded research"[All Fields] OR "grounded analysis"[All Fields] OR "life stor*"[All Fields] OR "emic"[All Fields] OR "etic"[All Fields] OR "hermeneutic*"[All Fields] OR "heuristic*"[All Fields] OR "semiotic*"[All Fields] OR "data saturat*"[Title/Abstract] OR "participant observ*"[Title/Abstract] OR "social construct*"[All Fields] OR "postmodern*"[All Fields] OR "post structural*"[All Fields] OR "post structural"[All Fields] OR "poststructural*"[All Fields] OR "post modern*"[All Fields] OR "interpret*"[All Fields] OR "action research"[All Fields] OR "cooperative inquir*"[All Fields] OR "humanistic"[All Fields] OR "existential"[All Fields] OR "experiential"[All Fields] OR "paradigm*"[All Fields] OR "field research"[Title/Abstract] OR "human science"[Title/Abstract] OR "biographical method"[Title/Abstract] OR "account"[All Fields] OR "accounts"[All Fields] OR "unstructured"[All Fields] OR "open ended"[All Fields] OR "text*"[All Fields] OR "narrative*"[All Fields] OR "life world"[All Fields] OR "conversation analysis"[All Fields] OR "personal experience*"[All Fields] OR "life experience*"[All Fields] OR "cluster sampl*"[All Fields] OR "theme*"[All Fields] OR "thematic"[All Fields] OR "categor*"[All Fields] OR "observational method*"[All Fields] OR "focus group*"[All Fields] OR "questionnaire*"[All Fields] OR "constant comparative"[All Fields] OR "constant comparison"[All Fields] OR "thematic analysis"[All Fields] OR "heidegger*"[Title/Abstract] OR "colaizzi*"[Title/Abstract] OR "speigelberg*"[Title/Abstract] OR "van manen*"[Title/Abstract] OR "van kaam*"[Title/Abstract] OR "merleau ponty*"[Title/Abstract] OR "husserl*"[Title/Abstract] OR "giorgi*"[Title/Abstract] OR "foucault*"[Title/Abstract] OR "corbin strauss*"[Title/Abstract] OR "strauss corbin*"[Title/Abstract] OR "glaser*"[Title/Abstract] OR "interview*"[All Fields] OR "qualitative"[All Fields])
