# Supplementary material for: Health care workers’ experiences during the COVID-19 pandemic: a scoping review
Source: Hum Resour Health. 2022 Mar 24;20:27. doi: 10.1186/s12960-022-00724-1 (PMC8943506; doi:10.1186/s12960-022-00724-1)
Supplement: Supplementary file 3 — Additional file 3: Table S3. List of countries studied. The file includes a table listing the countries in which the included studies were conducted according to frequency. [file 12960_2022_724_MOESM3_ESM.docx]

**Health Care Workers’ experiences during the COVID-19 Pandemic: a scoping review**

Additional file (3): List of countries represented in the included studies

| **Country** | **Frequency** |
| --- | --- |
| USA | 26 |
| China | 20 |
| UK | 12 |
| Canada | 11 |
| Iran | 8 |
| Spain | 7 |
| Italy | 7 |
| Turkey | 6 |
| Australia | 5 |
| Germany | 5 |
| Ireland | 5 |
| Pakistan | 5 |
| Brazil | 4 |
| India | 3 |
| Denmark | 2 |
| France | 2 |
| Israel | 2 |
| Lebanon | 2 |
| Netherlands | 2 |
| Nigeria | 2 |
| Argentina | 1 |
| Belgium | 1 |
| Ethiopia | 1 |
| Hong Kong | 1 |
| Indonesia | 1 |
| Japan | 1 |
| Jordan | 1 |
| New Zealand | 1 |
| Norway | 1 |
| Oman | 1 |
| Palestine | 1 |
| Slovenia | 1 |
| Senegal | 1 |
| Singapore | 1 |
| South Korea | 1 |
| South Africa | 1 |
| Zimbabwe | 1 |
| Multiple | 8 |
