# Supplementary material for: Health care workers’ experiences during the COVID-19 pandemic: a scoping review
Source: Hum Resour Health. 2022 Mar 24;20:27. doi: 10.1186/s12960-022-00724-1 (PMC8943506; doi:10.1186/s12960-022-00724-1)
Supplement: Supplementary file 4 — Additional file 4: Table S4. Detailed information on FGDs. This document provides information extracted from studies that used FGDs as a qualitative data collection tool. The table lists the overall number of focus group discussion’s participants in each of those studies, the number of FGDs per study, whether FGDs were conducted online or offline, the type of study participants, and any other information on the methods that could be extracted. [file 12960_2022_724_MOESM4_ESM.docx]

**Health Care Workers’ experiences during the COVID-19 Pandemic: a scoping review**

Additional file (4): Detailed information on FGDs

This document provides information extracted from studies that used FGDs as a qualitative data collection tool. The table lists the overall number of FGD’s participants in each of those studies, the number of FGDs per study, whether FGDs were conducted online or offline, the type of study participants, and any other information on the methods that could be extracted.

| **#** | **n. participants** | **n. FGDs** | **Mode** | **HCWs group** | **Qualitative methods** | **Comments** |
| --- | --- | --- | --- | --- | --- | --- |
| 1 | 30 | n.a* | In person | General Surgery residents | Only FGDs |  |
| 2 | 20 | n.a* | In person | Infection control nurse leaders | Only FGDs | . Each FGD lasted 60 to 90 minutes. |
| 3 | 7 | 1 | Online | Junior Physicians | Only FGDs | . The FGD was conducted following a quantitative survey.  . The one-hour virtual session took place via Zoom.  . The FGD covered 9 questions, which were based on survey responses. |
| 4 | 7 | 1 | Online | Community healthcare workers leaders | Only FGDs | . FGDs took place during a monthly meeting, with time at the beginning of the meeting for National Association of Community Health Workers (NACHW) state ambassador business.  . A trained moderator conduced the 90-minute FGD accompanied by a note-taker. |
| 5 | 24 | 4 | In person | Nurses | Only FGDs | . Six nurses, on average, participated in each FGD.  . Two researchers conducted the FGDs, one as moderator and one as interviewer  . All FGDs were conducted in a ventilated, quiet and private room in the hospital. All adhered to physical distancing and wearing of face masks. |
| 6 | 8 | 1 | In person | Nurses and medical secretaries at GP practice | Only FGDs | . The FGD lasted one hour at the medical centre |
| 7 | 40 | 6 | In person | HCWs involved in the management of COVID-19 at different managerial levels  Stakeholders working at the frontline in the management of COVID-19 (managerial, public health/field/community and primary care health centers) | Only FGDs | . Different locations were used, including the regional COVID-19 operational center (3 FGDs), the directorate of health services (2 FGDs), and a health center (1 FDG).  . A semi-circle sitting arrangement was set (1.5-2 meter-distancing).  . All participants were asked to wear masks and adhere to the infection prevention measures.  . Discussions were led by a trained facilitator and an assistant facilitator taking notes. |
| 8 | 39 | n.a* | online | Administrative leaders, physicians, nurses and medical support staff | Only FGDs | . Participants were offered the opportunity to  attend 1 of 7 virtual FGDs using the Zoom videoconferencing platform.  . Each FGD lasted one hour.  . A maximum of 10 participants in each FGD.  . FGDs were proceeded by a quantitative survey. |
| 9 | 34 | 4 | In person | FGD−1: Doctors  FGD-2: Nurses, Allied health professionals  FGD-3 Field staff (Community health workers, Social workers)  FGD-4: Drivers and Housekeeping staff | FGDs, Interviews and observation | . Duration of FGDs ranged from 45 to 60 min. |
| 10 | 32 | n.a* | Online | Consultant obstetrician/gynaecologist  Consultant psychiatrist, Consultant anaesthetist, Doctor, unspecified, General practitioner, Specialty trainees, Specialist nurse, Research nurse, Physiotherapist, Occupational therapist, Midwife, Dietician, Management/administration | FGDs and Interviews | . Participants were invited to tell their stories uninterrupted. |
| 11 | 22 | 5 | In person | Nursing staff (Some from the same department and others were mixed). | FGDs and observation | . The method followed a hospital ethnographic approach.  . Each FGD lasted on average 2 hours and included three to seven informants. |

*Not Available

**# Articles title**

1. Addressing General Surgery Residents’ Concerns in the Early Phase of the COVID-19 Pandemic
2. Barriers to using personal protective equipment by healthcare staff during the COVID-19 outbreak in China
3. Experiences of early graduate medical students working in New York hospitals during the COVID-19 pandemic: a mixed methods study
4. Insights on COVID-19 From Community Health Worker State Leaders
5. Nurses’ experiences of being recruited and transferred to a new sub‐intensive care unit devoted to COVID‐19 patients
6. Restructuring in a GP practice during the COVID-19 pandemic – a focus-group study
7. The Experiences and Perceptions of Health-Care Workers During the COVID-19 Pandemic in Muscat, Oman: A Qualitative Study
8. A National Study of Community Health Centers' Readiness to Address COVID-19
9. Challenges, experience and coping of health professionals in delivering healthcare in an urban slum in India during the first 40 days of COVID-19 crisis: a mixed method study
10. Developing services for long COVID: lessons from a study of wounded healers
11. We are not heroes—The flipside of the hero narrative amidst the COVID19‐pandemic: A Danish hospital ethnography
